# Web-based chatbot for Frequently Asked Queries (FAQ) in Hospitals

**DOI:** 10.1016/j.jtumed.2021.06.002

**Published:** 2021-07-04

**Authors:** Mamta Mittal, Gopi Battineni, Dharmendra Singh, Thakursingh Nagarwal, Prabhakar Yadav

**Affiliations:** a Department of Computer Science and Engineering, Delhi Skill & Entrepreneurship University, Okhla Campus-I, New Delhi, India; b Informatics in Medicine, School of Medicinal and Health Products Sciences, University of Camerino, Camerino, Italy

**Keywords:** روبوت المستشفى, البرمجة اللغوية العصبية, استخراج البيانات, تقنيات الويب, معلومات المستشفى, Bagging, Hospital bot, Natural language processing, Neural networks, Query, Web technologies

## Abstract

**Objectives:**

Local hospitals are operated by the resigned association of patients as passive communication channels. The online hospital data related to the users’ queries are not transparent and reliable. Therefore, it is crucial to have an intelligent web chatbot that manages user requests and provides quick access to local hospital information. In this paper, we present a framework and functionality of a chatbot developed using web technologies.

**Methods:**

The bot engine was integrated by several machine learning approaches like gradient descent (GD) and natural language processing (NLP) algorithms. The trained data entered into the bot were split into mini-word batches, and the GD algorithm was applied sequentially on each mini-batch. The NLP methods involved in converting a word to its stem with a text result less readable by humans.

**Results:**

The employed ML algorithms were successfully incorporated to manage the alternative synchronisation of text and voice messages.

**Conclusions:**

The proposed bot can be a better solution for data extraction from local hospital which functioning as a good communication channel for both users and hospital staff and helpful in reducing the crowd.

## الملخص

**أهداف البحث:**

تدار المستشفيات المحلية من قبل جمعية المرضى المستقيلة كقنوات اتصال سلبية. بيانات المستشفى عبر الإنترنت المتعلقة باستفسارات المستخدمين ليست مضمونة من حيث الشفافية والموثوقية. لذلك، من الضروري أن يكون لديك روبوت محادثة ذكي على الويب يمكنه استكشاف إدارة طلبات المستخدم، وتوفير وصول سريع لمعلومات المستشفى المحلي. في هذه الورقة، قدم المؤلفون إطار عمل ووظيفة روبوت محادثة تم تطويره بواسطة خبراء في تقنيات الويب.

**طرق البحث:**

تم دمج محرك الروبوت من خلال العديد من مناهج التعلم الآلي مثل خوارزميات الانحدار ومعالجة اللغة الطبيعية. وتم تقسيم البيانات المدربة التي تم إدخالها في الروبوت إلى مجموعات من الكلمات المصغرة وتم تطبيق خوارزمية الانحدار على كل دفعة صغيرة بالتتابع. طرق البرمجة اللغوية العصبية المتضمنة في تحويل الكلمة إلى جذعها مع نتيجة نصية منخفضة يمكن قراءتها من قبل البشر.

**النتائج:**

تم دمج الخوارزميات المستخدمة بنجاح لإدارة المزامنة البديلة للرسائل النصية والصوتية.

**الاستنتاجات:**

يمكن أن يكون الروبوت المقترح حلاً أفضل لاستخراج البيانات من المستشفيات المحلية. إنها قناة اتصال ثاقبة لكل من المستخدمين وموظفي المستشفى.

## Introduction

A chatbot is a type of software that can communicate with humans. It is integrated with Artificial Intelligence (AI) techniques such as machine learning (ML) and natural language processing (NLP).^1^ These conversational agents are specially designed as a user interface (UI) that engages as a communication channel. Currently, chatbots are integrated into every business, including hospitals, banks, transport, and other services. There is a great demand for them to work as information agents providing instant responses in the healthcare industry.

Hospitals or healthcare organisations manage patient communications through rudimentary web operations.^2^ Web-based chatbots overcome this by allowing conversations between hospital staff and patients to collect insights on facilities available in the hospital.^3^ Chatbots have been receiving close attention in healthcare because these conversational agents provide easy access to information and function as an interactive platform offering personal engagement.^4^ Medical chatbots provide the necessary information by including classification and ML algorithms.^5^ The chatbot developed in^6^ has successfully maintained responses for disease predictions, medication recommendation, doctor availability, and others by adopting simple ML techniques. Additionally, web-based chatbots based on NLP can assess patient sentiments and improve quality care.^7^

Additionally, the other two popular conversational agents, ELIZA and A.L.I.C.E (Artificial Linguistic Internet Computer Entity), are known as the first human interaction chatbots. ELIZA operates using computational linguistics with simply parsing and keyword substitution into reframed phrases.^8^ Therefore, it can understand user experiences and feelings. This conversational agent initiates the user chat with simple sentence structure and punctuation, and the user statement is ended with a twofold carriage response. However, A.L.I.C.E can convert readable texts (i.e. corpus) into Artificial Intelligence Markup Language (AIML) to disclose the possibility of useful prototypes without complex ML algorithms.^9^ It also incorporates NLP techniques that help engage in human conversation by applying heuristically matched rules to the user input. It was inspired by the old ELIZA program.

However, these existing systems failed to save the chat history and were not fully customised to understand the user's ultimate message. Thus, these bots did not fully understand what the user said and provided responses from the knowledge stored in the bot brain. Therefore, we developed a special chatbot that includes collecting local hospital information responses by integrating web-based techniques. Present medical chatbots are integrated with speech recognition such that users can communicate through either voice or text messages.

The major objective of this work is to explain the importance of medical chatbots and present our developed medical chatbot, developed on internet technologies. Our chatbot also assists user queries regarding hospital information, including specialists' availability, OPD timings, room registration, number of beds, emergency information, and doctor availability, among others. This is the first real-time developed medical chatbot for query queue management in hospitals based on the literature survey. Additionally, it improves users’ satisfaction by providing answers to all their health and personal assistance related queries. The proposed chatbot virtually assists users like real reception staff of a hospital. It provides users with total medical assistance 24∗7.

## Materials and Methods

### Gradient descent algorithm

To implement a chatbot, several techniques and optimisation algorithms are available. Gradient Descent (GD) is an optimisation algorithm used to evaluate the coefficients of function (f) that minimise the cost function.^10^ It is a primary optimisation algorithm to assess the minimum cost function. The presented bot adopts a mini-batch GD algorithm. By applying the GD algorithm, the model can be accommodated easily in memory with low noise. For instance, if the trained set has 100 training examples, then it is divided into five batches with each batch of 20 training examples, explained as follows:

*The GD procedure*

• The algorithm starts with some small random values (possibly 0.0) of the function. i.e., coefficient = 0.0
• The coefficient costs are calculated by inserting them into the function. i.e., cost = f (coefficient).
• The derivative cost can be calculated to assess the lower cost in further iterations. i.e., Delta (∇) = derivative (cost).
• A learning parameter (α) is introduced to control the coefficient change for every update.

Coefficient = coefficient−(α∗∇)

• These steps are repeated until the cost of the coefficients is nearly equal to zero.

By combining these steps, the mini-batch GD function can be presented as,

$$\theta =\theta -\eta .\;\nabla \;\theta J\;\left(\theta \text{;}{\text{x}}^{\left(\text{i;i}+\text{n}\right)}\text{; }{\text{y}}^{\left(\text{i;i}+\text{n}\right)}\right)$$

Accordingly, by integrating with different machine learning algorithms such as GD, NLP, and feed-forward neural network (FNN), the chatbot can produce accurate output responses. The description of each algorithm has been discussed in further sections.

### Natural language processing techniques

NLP is an integral part of AI that deals with interactions between human languages and computers. Stemming is a sub-stream of NLP techniques, which reduces a word to its stem with a text result, less readable by humans. It is an algorithm that converts the word into a root word.^11^ In addition to GD, text classification algorithms are included to evaluate better outcome response. Initially, the user inputs are applied to the NLP framework to generate an output response. The input undergoes different steps such as tokenisation, stemming, and enumeration and converted to a bag of words. In tokenisation, simple tokens are broken into simple words. Next, the tokens are transferred as root words; this is called stemming. The bag of words model pre-processes the given text by converting its simple words and keeps a count of total occurrences of high-frequency words. Finally, the bag of words produces the input for the ANN model and evaluates individual probabilities of each response to generate the highest probability words as outcomes fetched from the JavaScript object notation (JSON) file.

### Text to speech/Speech to text conversation

The text to speech (TTS) system converts natural human language text into speech.^12^ Speech is a general way of communication between human beings. Texting and voice are becoming a major bot interface. Soon there will be high demand for speech-enabled chatbots. The TTS system integrated with the proposed chatbot comprises two parts, a front-end and a back-end. TTS converts raw text to numbers and abbreviations into the equivalent of text out words in the front end. Additionally, it assigns phonetic transcriptions to each word and divides the given text into prosodic units like phrases, clauses, and sentences. Finally, the back-end converts the symbolic linguistic representation into sound.

The speech to text (STT) system is trained to accept input like human speech to interpret and transcribe into text. The proposed chatbot has inbuilt technology to break long sentences or words into distinct phonemes. It works based on complex algorithms to create precise text and presents the user's speech to the bot, transforming verbal speech into understandable text.^13^

## Results

The working of a web-based medical chatbot, majorly associated with two functional operations, included word prediction and pattern matching. In this section, the authors present a comprehensible explanation of those operations.

### Functionality operations

#### Pattern matching behaviour of testing outcomes

The pattern matching algorithm followed by the presented bot is similar to that of the ALICE bot system. The employed AIML interpreter matches each word to large patterns to determine the best fit. We tested the bot with different categories of commands to verify the response. Here are four category examples of the commands supplied to the bot.

**Table:** 

(1)	<category>
	<pattern> HOW ARE YOU DOING TODAY </pattern>
	<template><sr/> <srai> HOW ARE YOU DOING TODAY</srai></template>
	</category>
(2)	<category>
	<pattern> HOW ARE YOU DOING </pattern>
	<template> <random> <li> GOOD</li><li>NOT SO </li><li> WELL </li><li> NOT BAD</li></random></template></category>
(3)	<category>
	<pattern> HELO </pattern>
	<template> <srai>HELLO</srai></template>
	</category>
(4)	<category>
	<pattern> HELLO </pattern>
	<template> <random> <li>HI THERE! </li><li>WELL HELLO THERE! </li> <li> HELLO THERE </li><li> HI THERE, CAN I TALK TO YOU </li></random></template>
	</category>
	User Input: HELO HOW ARE YOU DOING TODAY
	Bot response: HI THERE! GOOD.

**Figure 1:**
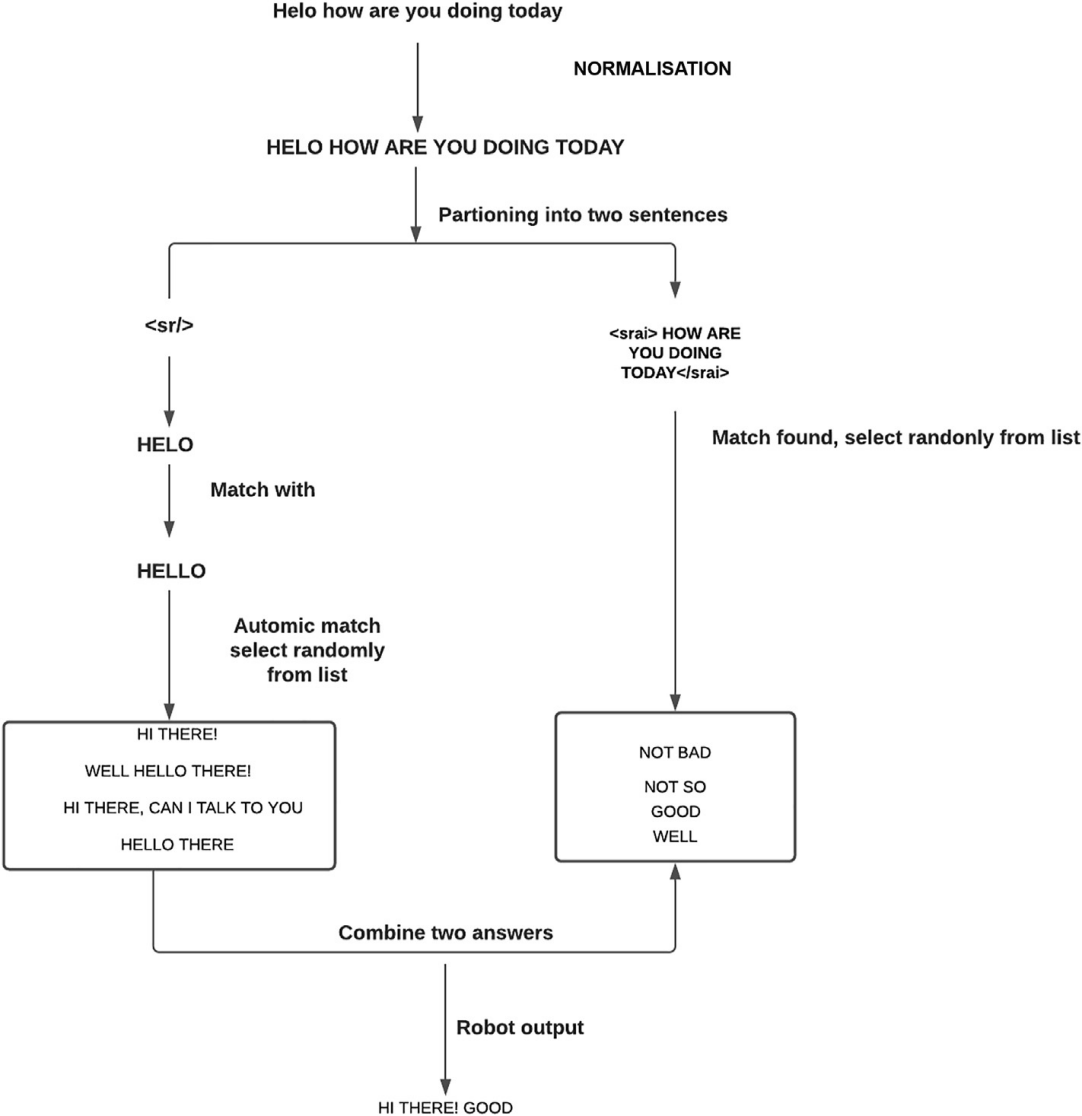
The tree structure of pattern matching behaviour.

Figure 1 depicts the tree structure pattern matching procedure.

#### Word prediction model development

We used a fairly standard model of a feed-forward neural network with two hidden layers. FNN is an artificial neural network in which information moves in only one direction, forward from the input nodes, via the hidden nodes, and to the output nodes. The goal of this network is to assign a class to each word from a bag of words (one of the tags from the JSON file). The trained data are split into small word batches, and the GD algorithm is applied to every batch sequentially to find the appropriate response. It makes the algorithm function faster and efficiently. Only a single batch of words can be passed through the neural networks at a time to compute the loss of every sample batch. Their average is used to update the neural network parameters. The mini-batch descent algorithm finds the best of two words and updates each mini-batch of n_n_ training samples. In a given command, the trained set has 1000 training examples. It is divided into eight batches, with each batch comprising 125 training examples. Model training is conducted using the number of epochs, set according to the number of times that the model will encounter the same information while training. The training command of the model is:

**Table:** 

*model.fit (training, output, n_epoch=1000, batch_size=8, show_metric=True)*
*model. save ("model.tflearn")*

**Figure 2:**
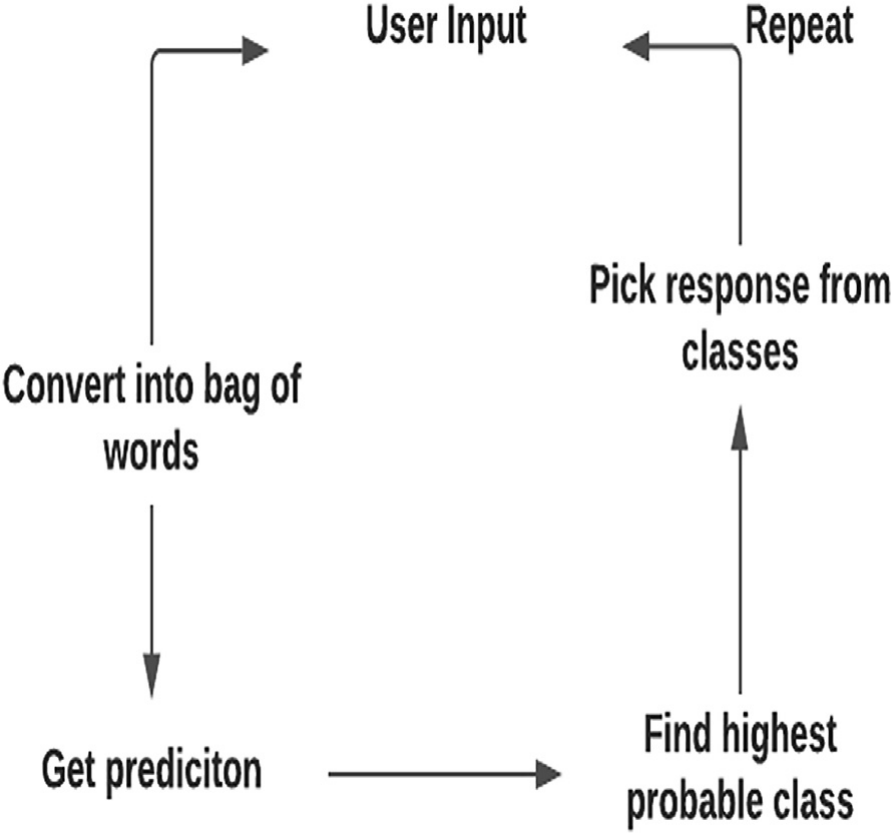
Word prediction model.

The outcome model is saved as a *model.tflearn* to include future scripts. This model is trained by the mini-batch GD algorithm. The model predictions are obtained by feeding the bag of words based on the high probability words selected. Thus, the process generates a response, as shown in Figure 2. Next, the chat function obtains a prediction from the model and grabs an appropriate response from the given JSON file.

### Bot designing steps and architecture

The designed chatbot in the study engages with users via a microphone and simultaneous translation of voice to text and text to voice using deep neural networks, producing the relevant matched text response (output). The step-by-step designing of the chatbot has been discussed below.

➢ The user starts the conversation by selecting a special code number of a particular hospital. Once the hospital is selected, the corresponding JSON file of that hospital is loaded, including all queries requested by the user related to that hospital. A JSON file with the predefined tags, patterns, and responses encompassing the bot functionality is also included. Integrating with the Google application programming interface (API), the bot can simultaneously respond to voice and text conversations.
➢ In the second step, users' queries are accepted through the microphone. Here, the microphone can be in-built or external and can work as a data transmission source. The web-kit speech recognition API is used to recognise the user input, voice or text.
➢ In the third step, the input voice is converted into corresponding text using a web-kit speech recognition API. Subsequently, the text data can be analysed using special NLP algorithms like bagging.
➢ In the fourth step, the matched responses to the corresponding voice message are converted using gTTS (Google Text-to-Speech) API. Finally, the audio response output is played using the play sound function of the python module and is spoken out through the speaker.

In the end, users can decide whether to continue with the same hospital by providing the next set of queries or switch to another hospital by asking queries particular to it. If a user wants to continue with the same hospital, control moves to steps 2 to 4. However, if a user wants to switch to another hospital, a simple voice or text command can trigger step 1.

The user can ask any query through text or voice without being physically available at the hospital. Thus, the system can conduct a one-to-one conversation with patients and saves time instead of waiting in long queues. This section presents the functional framework of chatbots and NLP preliminary outcomes. The designed bot includes a speech recognition feature to decode voice input and output, stored in a database of the JSON files. Each hospital database is associated with multiple JSON files. When the user enquires about a hospital, the corresponding JSON file is triggered to present relevant responses. Each hospital has a unique identification number. Switching between different hospitals is possible using the command ‘change hospital’. At the front-end, the microphone, used as a speech input source, converts voice to text. This is implemented using python language, including play sound and gTTS modules. A user interface is created using a flask and HTML, CSS, AJAX, and JavaScript. Here, HTML and CSS are used to create the User Interface (UI) structure and page styling, while JavaScript is used to make the web page dynamic and interact with the main python code at the back-end.

**Figure 3:**
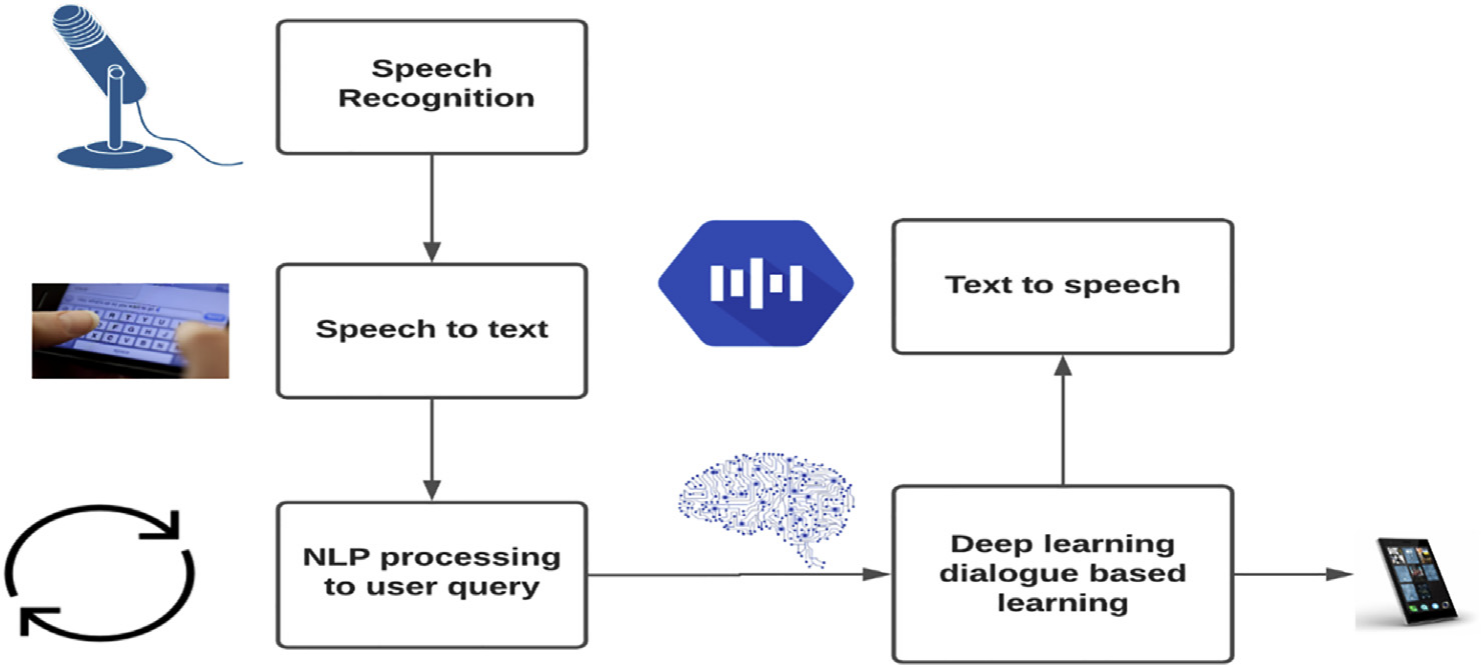
The functional framework for the proposed web-based chatbot

The web-based UI manages patient queries through either text or voice and displays the response.^14^ The user can use the microphone button to deliver voice messages. Based on the voice input system, the response is displayed. Subsequently, the user confirms the response using the send button. Simultaneously, users can use text input for inquiry and response outcomes generated in text and voice form. The architecture of the proposed web-based chatbot is depicted in Figure 3.

### Chatbot performance

The performance of the developed chatbot is defined based on two factors, accuracy and loss. Accuracy is defined as the correct intent returned for the percentage of speech. Overall accuracy is the number of correct predictions vs. the total number of predictions. The accuracy of a neural network depends on many factors, such as the activation function and the number of neurons, among others. We successfully obtained 100% accuracy from the 393rd iteration step for the chatbot trained for approximately 1000 epochs. The accuracy was consistent till the last iteration.

**Figure 4:**
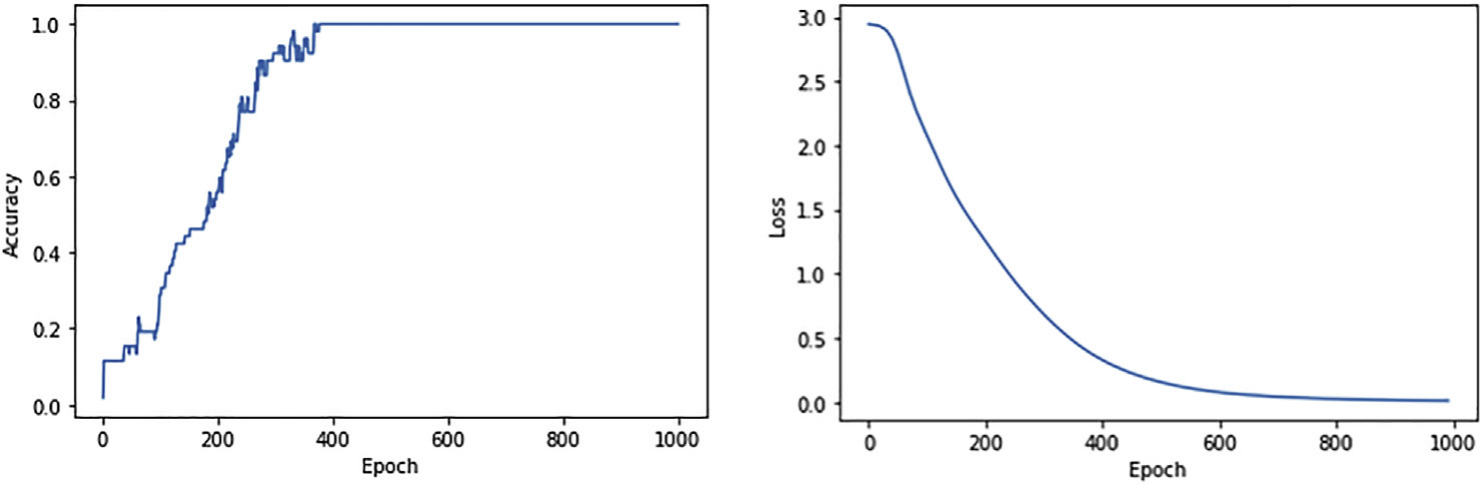
Overall chatbot performance curves.

Loss is defined as the percentage of prediction error in the neural network model. The loss function is an important component of neural networks. Loss is used to calculate the gradients and gradient update of the weights of the neural network. The loss function of our chatbot is decreasing from the first iteration and falls to 0 at the 697th iteration. The decrease in loss function starts from the 29th iteration and consistently declines till the last iteration. This proves the efficiency of our chatbot. Figure 4 represents the overall performance of the chatbot model developed.

## Discussion

Medical chatbots have become popular because of the new ways of communication between patients and doctors. For a decade, many health organisations have adopted these bots in their work. In emergency health conditions, every patient requires information regarding available local hospitals, including information about doctors’ availability, bed facilities, and ambulance services, among others. However, it is not easy to be physically present in emergencies.

Healthcare organisations are adopting modern knowledge through AI technology to manage patient feedback.^15^ Both AI and NLP are becoming more popular in the medical domain with the continuous advancement of information technology.^16^^,^^17^ Several computing developers have developed effective and efficient systems by including these techniques to save users time and provide accurate results.^17^ This study presents a designed chatbot application to manage all patient queries regarding different hospitals. The platform uses a training algorithm to train the chatbot depending on a bag of words, helping the patients find responses to corresponding queries. Additionally, the system automatically retrieves the updated hospital information with beds and doctor availability. Subsequently, it sends an input variable to the neural networks to compare other possibilities to recommend similar hospital facilities.

People fall ill at any time, and sometimes, it leads them to mortality. Accordingly, every user anticipates searching for the nearest hospital or health care centre, including better medical facilities. Instead of surfing for the individual hospital site, the present bot produced collective information of medical centres in a domestic region. The front-end of the web-based bot is developed using HTML and JavaScript languages. The engine integrated with NLP techniques behaves as back-end logic to understand user response via API calls.

The efficiency of the chatbot can be improved by adding more combinations of words and increasing the use of the database so that the medical chatbots can handle all types of queries.^18^ Even voice conversation and Speech Recognition can be added to the system for ease of use. Chatbots have become increasingly more popular in the past two years and are accepted widely. They bring new ways to communicate with humans and make communication interesting. Furthermore, the rise of technologies and AI provide convenience to users.^19^ Normally when a person visits the hospital, they first go to the help desk to collect information about the doctors, registration, and blood bank, among others. Our chatbot, Chatbot for Hospital Management, helps the users by providing all the necessary information about a hospital in just one click. It proves to be a great help to society as it helps avoid time-consuming long queues at the help desk in any hospital. Hence, our medical chatbot is more useful for different patients seeking various medical assistance.

There are some established medical chatbots like Health Online Medical Suggestion (HOLMeS) and IBM Watson.^20^ Both bots are well known in the medical industry for their deep learning and NLP approaches. However, these systems are not well developed for word prediction models and pattern matching behaviours. Instead, they create a smooth interaction with the patient to help them with supplied knowledge. Thus, they limit the ability to generate new knowledge.^21^ However, the designed bot has a special feature of voice recognition, a better graphical interface, and emergency information of city hospitals. When the patient is in a serious condition, this system allows the user to check for hospitals with the appropriate medical opportunities, including emergency. Additionally, it can assist hospital staff to display user responses for particular queries. This proposed web application can allow users to develop an idea about the medical facilities of local hospitals. Moreover, the user can alternate between hospitals using the command ‘change hospital’ and inquire about that particular hospital. NLP techniques like stemming and tokenisation are employed to generate an explicit response.

The present system is a speech recognition based chatbot related to health care. Here, users can request queries through a microphone, and the speech is translated into text using NLP. Then, this text is analysed through deep neural networks to match the relevant response in the corresponding JSON files (the user can select any hospital of his/her own choice, and then that particular JSON file gets loaded). Thereafter, the matched outcome text will be converted into audio, supplied as input to the microphone.

Despite the advantages, the developed bot has some limitations. First, even if the response is quick, there are limited responses to users’ questions. Second, setting up AI on a large scale and testing can be expensive for hospital authorities. However, the bots can learn automatically with little time consumption. Additionally, hospitals aim to provide better healthcare services, reliable data, and good quality care. Thus, information sharing must be transparent between the service provider and patient. However, ensuring reliable information is updated into the standardised medical database would add extra workload to hospital management staff. Therefore, automated data update frameworks have to be incorporated, and presently, no such methods are available.

## Conclusions

In this work, the authors presented a patient-friendly chatbot on hospital management, designed to provide all the important information of a hospital in simply one click. The system includes natural language processing methods to interpret human language such that it can identify grammatically incorrect or incomplete sentences using a similar bag of words approach. The chatbot is user friendly and can be used by any person who knows how to type in their language in a computer. Additionally, the medical chatbot provides the details of the availability of doctors. In the future, the symptom recognition and diagnosis performance could be greatly improved by more medical features, such as location, duration, and intensity of symptoms, and more detailed symptom descriptions. Thus, the medical chatbot has a vast future scope. No matter how far people are, they can have access to medical facilities.

## Source of funding

This research did not receive any specific grant from funding agencies in the public, commercial, or not for profit sectors.

## Conflict of interest

The authors have no conflict of interest to declare.

## Ethical approval

The authors did not have permission for direct communication with a human participant in the study, and no ethical issues were encountered during the study presentation.

## Authors' contribution

MM and GB conceived and designed the study, conducted research, provided research materials, and collected and organised data; DS: analysed and designed the framework; TSN, GB, and PY Wrote the initial and final drafts of the article and provided logistic support. All authors critically reviewed and approved the final draft and are responsible for the manuscript's content and similarity index.
